# Advances in Quantum Computation in NISQ Era

**DOI:** 10.3390/e27101074

**Published:** 2025-10-15

**Authors:** Xu-Dan Xie, Xiaoming Zhang, Balint Koczor, Xiao Yuan

**Affiliations:** 1 School of Physics, South China Normal University, Guangzhou 510631, China; 2 Mathematical Institute, University of Oxford, Woodstock Road, Oxford OX2 6GG, UK; 3 Center on Frontiers of Computing Studies, Peking University, Beijing 100871, China

## 1. Introduction

Realizing a universal, fault-tolerant quantum computer remains challenging with current technology. In the meantime, a more pressing and timely question is what can realistically be accomplished with existing and near-term quantum hardware in the noisy intermediate-scale quantum (NISQ) era. Leveraging the paradigm of hybrid quantum–classical computing, recent studies have showcased the potential of NISQ devices in diverse domains, including quantum chemistry, materials science, many-body physics, and machine learning. Yet, the inherent limitations of NISQ hardware—such as restricted qubit numbers, imperfect gate fidelity, and limited qubit connectivity—impose fundamental obstacles. Consequently, achieving a definitive quantum advantage over classical methods for practical problems remains unresolved. At the same time, there is a pressing need for new theoretical frameworks capable of rigorously benchmarking the performance and capabilities of NISQ devices under realistic constraints.

This Special Issue focuses on recent theoretical and experimental advances in quantum computing in the NISQ era. It includes both original research articles and comprehensive reviews covering, but not limited to, the following areas:

- Design of More Efficient Variational Quantum Algorithms: Variational quantum algorithms (VQAs) constitute a central paradigm of NISQ computing [1], yet their performance is often constrained by hardware noise, limited circuit depth, and optimization challenges. Current research seeks to develop hardware-efficient ansatz structures, adaptive circuit designs, and problem-inspired parameterizations to enhance expressivity and scalability. These efforts aim to reduce resource requirements while improving convergence properties and mitigating barren plateau issues.
- Applications in Chemistry, Materials, and Other Physics Problems: Quantum simulation remains one of the most promising domains for NISQ devices [2]. By targeting electronic structure problems, many-body dynamics, and condensed matter models, researchers are developing tailored algorithms that exploit physical insights for efficient circuit design. Advances in this direction may pave the way toward near-term quantum advantage in areas such as materials discovery, catalysis, and energy science [3].
- Applications in Machine Learning, Combinatorial Problems, and Beyond Physics: NISQ devices are being increasingly explored in domains that extend beyond traditional physics. Areas such as quantum machine learning [4], combinatorial optimization [5], and data-driven methodologies present rich opportunities for research. A key step toward demonstrating real-world impact lies in identifying classes of problems where quantum models provide inherent structural advantages over classical approaches.
- Analysis of the Performance of Hybrid Quantum–Classical Algorithms: Hybrid algorithms leverage classical optimization to guide quantum circuits, but their efficiency critically depends on noise resilience, optimization landscapes, and hardware–algorithm co-design [6]. Systematic performance analyses—such as studying scaling behavior under noise and benchmarking against classical baselines—are essential for identifying regimes where hybrid approaches can deliver genuine advantages.
- Theoretical Tools for Studying Ansatz Expressivity and Trainability: Understanding the representational capacity of variational circuits and the trainability of their parameters is fundamental for predicting algorithmic performance [7]. Emerging theoretical frameworks, drawing from quantum information theory, statistical physics, and optimization theory, aim to characterize expressivity, quantify entanglement generation, and analyze gradient landscapes. These developments provide principled guidance for the design of effective VQAs.
- Quantum Error Mitigation: As large-scale error correction remains beyond current technological capabilities, error mitigation has become indispensable for improving the effective performance of NISQ devices [8]. Techniques such as zero-noise extrapolation [9], randomized compiling [10], and symmetry-based approaches [11] continue to evolve, offering practical strategies to enhance computational accuracy without excessive resource overhead.
- Quantum Error Correction: Despite its substantial resource demands, quantum error correction remains an indispensable route toward scalable and fault-tolerant quantum computation [12]. In the NISQ era, research efforts have emphasized lightweight codes, customized error-detection protocols, and proof-of-concept demonstrations of small logical qubits [13]. These developments lay the foundation for transitioning from noisy intermediate-scale devices to architectures that are resilient against errors.
- Benchmarking the Performance and Power of NISQ Devices: Assessing the practical capabilities of NISQ processors requires rigorous benchmarking. In addition to general-purpose metrics such as quantum volume, emerging task-specific benchmarks aim to quantify computational power for applications in chemistry, optimization, and machine learning [14]. Such frameworks are critical for identifying performance bottlenecks and guiding future developments in both hardware and algorithms.
- Experimental Realization of Variational Quantum Algorithms: Experimental demonstrations of VQAs on current platforms—including superconducting qubits, trapped ions, and photonic systems—provide crucial validation of theoretical concepts under realistic noise conditions [15,16]. These implementations also reveal practical challenges in circuit execution, parameter optimization, and measurement overhead. Continued progress in this direction will be pivotal for bridging the gap between theoretical promise and practical utility.

This Special Issue includes fourteen recent studies advancing quantum computing across algorithms, applications, and hardware. In physics applications, the first article [17] presents a quantum algorithm for simulating the Lorenz model, while the second [18] surveys superconducting quantum simulators for nonequilibrium many-body systems. In broader applications beyond physics, the third study [19] develops a hybrid quantum recurrent neural network for fraud detection, the fourth [20] applies QAOA to the Independent Domination Problem, and the fifth [21] combines branch-and-bound with quantum annealing for NP-hard problems, while the sixth [22] explores minimal quantum reservoir computing with Kerr oscillators. On the theoretical side, the seventh article [23] introduces tensor decision diagrams for quantum circuit equivalence. To improve the performance of the algorithm, the eighth article [24] investigates dynamical decoupling and circuit co-design on IBM processors, and the ninth [25] enhances Grover Adaptive Search with QAOA for constrained optimization. Hardware-oriented contributions include the tenth article [26], which proposes Leakage Randomized Benchmarking, and the eleventh [27], which studies dephasing effects on Grover’s algorithm. In error correction, the twelfth [28] introduces recursively expanded stabilizer codes achieving constant rate and Pauli error correction. Finally, in the pursuit of quantum advantage, the thirteenth article [29] evaluates Simon’s algorithm on NISQ devices, and the fourteenth [30] proposes a hybrid boson sampling scheme with photons and Bose–Einstein condensates.

In summary, this Special Issue is organized to provide a dedicated platform for presenting and discussing the latest developments in noisy intermediate-scale quantum (NISQ) computing. It seeks to bring together high-quality contributions that address emerging challenges and propose innovative solutions across a wide range of topics, including hybrid quantum–classical algorithms, quantum error mitigation, benchmarking of NISQ devices, and applications in chemistry, materials science, and machine learning.

## 2. An Overview of Published Articles

In the applications of physics problems, the first article [17] presents a quantum algorithm for simulating the second-order time-discretized Lorenz model—a benchmark system in climate science, fluid dynamics, and chaos theory. Its recursive design reduces initial-state copy requirements to linear scaling, improving efficiency over previous methods while maintaining quantum speed-up. Classical simulations verify its accuracy in reproducing both regular and chaotic attractors within relevant parameter regimes. The second article [18] reviews the applications of superconducting quantum simulators in nonequilibrium many-body physics, with a particular focus on recent experimental advances in exploring emerging nonequilibrium quantum phenomena such as many-body localization, quantum many-body scars, and discrete time crystals. It also discusses the prospects of quantum simulation experiments in addressing open problems of nonequilibrium many-body systems in the future.

In the context of machine learning, combinatorial optimization, and broader applications beyond physics, four recent studies illustrate complementary advances. The third [19] develops a hybrid quantum recurrent neural network (HQRNN-FD) for fraud detection, integrating variational quantum circuits with angle encoding, data reuploading, and hierarchical entanglement to enhance feature extraction. This approach achieves an accuracy of 0.972 on benchmark datasets—outperforming classical baselines—while maintaining robustness to quantum noise and scalability with qubit count. The fourth study [20] applies the Quantum Approximate Optimization Algorithm (QAOA) to the Independent Domination Problem (IDP), showing on IBM’s qasm_simulator that QAOA can outperform classical approaches under suitable parameter choices, thereby opening up new directions for quantum optimization of graph problems. The fifth work [21] introduces a hybrid classical–quantum framework that combines branch-and-bound with quantum annealing to solve NP-hard integer linear problems such as the Knapsack and Traveling Salesman Problems. By decomposing large-scale problems into QUBO instances solvable on D-Wave Advantage, this method balances classical and quantum resources, yielding solutions superior to random guessing and more scalable than standalone quantum annealing while highlighting trade-offs between solution quality and efficiency. Finally, the sixth study [22] investigates a minimal quantum reservoir computing (QRC) model of two coupled, driven–dissipative Kerr oscillators. Using Partial Information Decomposition, the authors show how dynamical instability and dissipation shape the balance between redundant and synergistic information encoding, with dissipation unexpectedly enhancing memory capacity by promoting redundancy. This work establishes a fine-grained, information–theoretic framework for QRC design and demonstrates that dissipation itself can serve as a computational resource.

Within the theme of theoretical tools for studying ansatz, the seventh article [23] tackles the quantum circuit equivalence problem by reformulating it as a tensor network contraction task. The authors introduce tensor decision diagrams (TDDs) for symbolic tensor representation, which exploit structural symmetries for efficient compression. The experimental results demonstrate that the TDD-based framework performs competitively in equivalence checking of quantum circuits.

To improve the performance of quantum algorithms, the eighth article [24] explores the synergistic effects of dynamical decoupling (DD) and optimized circuit design in enhancing algorithm performance on near-term quantum devices. Using eight IBM processors, the study analyzes how hardware features and algorithm design impact the effectiveness of DD for error mitigation. This study provides valuable insights for optimizing DD protocols and circuit architectures through a holistic hardware–algorithm co-design approach. The ninth article [25] proposes an enhanced Grover Adaptive Search (GAS) algorithm to address the Constrained Polynomial Binary Optimization (CPBO) problem. By leveraging the Quantum Approximate Optimization Algorithm (QAOA) to refine initial threshold selection, the method mitigates limitations of standard GAS and achieves faster convergence. The experimental results on Max-Cut and CPBO instances demonstrate significant improvements in algorithmic acceleration.

In the study of noise characterization, the tenth article [26] introduces Leakage Randomized Benchmarking (LRB), an efficient framework for measuring leakage rates in multi-qubit systems, which is insensitive to state preparation and measurement (SPAM) noise. The protocol extends to an interleaved variant (iLRB) for benchmarking average leakage rates of generic n-site quantum gates. This work provides a practical framework for diagnosing leakage errors, which is essential for the development of scalable quantum hardware. Complementing this hardware-level perspective, the eleventh article [27] analyzes the impact of pure dephasing noise on Grover’s quantum search algorithm. Implemented in a 4-qubit simulated environment using the Atos Quantum Assembly Language (AQASM) and the myQLM toolkit, the study shows that dephasing noise significantly reduces algorithmic accuracy: as the dephasing time decreases, the probability of successfully identifying the target state drops sharply. Nevertheless, the results demonstrate that AQASM and myQLM are effective tools for simulating noise effects in quantum hardware.

In the domain of quantum error correction, the twelfth article [28] proposes a new direction for designing high-rate quantum stabilizer codes by recursively expanding the Tanner graph of existing stabilizer codes, inspired by the recursive structure of classical polar codes but applied differently. Following this approach, the authors successfully design a class of quantum stabilizer codes achieving a constant coding rate of 0.5, capable of correcting two Pauli errors, offering a promising alternative path beyond the limitations of quantum polarization-based methods.

In the study of quantum advantage, the thirteenth article [29] examines the performance of Simon’s algorithm on NISQ devices and benchmarks the error rates of cloud-based quantum hardware. It finds that algorithmic error rates increase with problem size across all the tested devices, revealing that current NISQ platforms remain incapable of achieving a genuine quantum advantage. This study further highlights the critical role played by the quantum processing unit (QPU) architecture when transpiling algorithms onto physical platforms. The fourteenth article [30] proposes a hybrid boson sampling method, in which coupled photon and Bose–Einstein condensate atom systems are placed inside a multimode cavity to perform sampling, thereby testing quantum advantage over classical computers. This study shows that the joint probability distribution of this hybrid sampling scheme is #P-hard, indicating its potential for demonstrating a quantum advantage.

## 3. Conclusions

Overall, the collected works highlight both the rapid progress made and the persistent challenges of quantum computing in the NISQ era. They demonstrate how theory, algorithms, hardware development, and noise management are advancing in parallel, while also underscoring that achieving a true fault-tolerant quantum advantage remains a long-term objective. Together, these studies capture a field in dynamic transition, one that is simultaneously pushing the limits of NISQ devices while laying the essential groundwork for the next generation of scalable quantum technologies.
